# Dietary Supplementation With *Acer truncatum* Oil Promotes Remyelination in a Mouse Model of Multiple Sclerosis

**DOI:** 10.3389/fnins.2022.860280

**Published:** 2022-05-02

**Authors:** Yuhuan Xue, Xiaoyan Zhu, Wenyong Yan, Zhihan Zhang, Enhui Cui, Yongji Wu, Cixia Li, Jiarong Pan, Qijiang Yan, Xuejun Chai, Shanting Zhao

**Affiliations:** ^1^College of Veterinary Medicine, Northwest A&F University, Yangling, China; ^2^Multiple Sclerosis Research Center of New York, New York, NY, United States; ^3^Department of Basic Medicine, Xi’an Medical University, Xi’an, China

**Keywords:** *Acer truncatum* oil, neurodegenerative disease, demyelination, remyelination, cuprizone

## Abstract

**Background:**

Multiple sclerosis is a chronic demyelinating disease of uncertain etiology. Traditional treatment methods produce more adverse effects. Epidemiological and clinical treatment findings showed that unknown environmental factors contribute to the etiology of MS and that diet is a commonly assumed factor. Despite the huge interest in diet expressed by people with MS and the potential role diet plays in MS, very little data is available on the role of diet in MS pathogenesis and MS course, in particular, studies on fats and MS. The oil of *Acer truncatum* is potential as a resource to be exploited in the treatment of some neurodegenerative diseases.

**Objective:**

Here, we investigated the underlying influences of *Acer truncatum* oil on the stimulation of remyelination in a cuprizone mouse model of demyelination.

**Methods:**

Cuprizone (0.2% in chow) was used to establish a mouse model of demyelination. *Acer truncatum* oil was administrated to mice during remyelination. Following techniques were used: behavioral test, histochemistry, fluorescent immunohistochemistry, transmission electron microscope.

**Results:**

Mice exposed to cuprizone for 6 weeks showed schizophrenia-like behavioral changes, the increased exploration of the center in the open field test (OFT), increased entries into the open arms of the elevated plus-maze, as well as demyelination in the corpus callosum. After cuprizone withdrawal, the diet therapy was initiated with supplementation of *Acer truncatum* oil for 2 weeks. As expected, myelin repair was greatly enhanced in the demyelinated regions with increased mature oligodendrocytes (CC1) and myelin basic protein (MBP). More importantly, the supplementation with *Acer truncatum* oil in the diet reduced the schizophrenia-like behavior in the open field test (OFT) and the elevated plus-maze compared to the cuprizone recovery group. The results revealed that the diet supplementation with *Acer truncatum* oil improved behavioral abnormalities, oligodendrocyte maturation, and remyelination in the cuprizone model during recovery.

**Conclusion:**

Diet supplementation with *Acer truncatum* oil attenuates demyelination induced by cuprizone, indicating that *Acer truncatum* oil is a novel therapeutic diet in demyelinating diseases.

## Introduction

Multiple sclerosis (MS) is a frequent idiopathic immune-mediated demyelinating disease (Barnett and Prineas, 2004; Longo et al., 2018). This disease affects 2–3 million people worldwide and most of them are young adults in western countries, especially Europe and United States (Filippi et al., 2018). The underlying etiology of MS remains enigmatic (Clanet, 2008). What is clear, however, is that demyelination in the central nervous system and inflammatory responses are hallmarks of MS (Frischer et al., 2009; Stys et al., 2012). Extensive studies have revealed the involvement of genetic and environmental factors in the pathogenesis of MS. The environmental factors contributing to MS include stress (Brown et al., 2006), a bad climate (Norman et al., 1983; Laborde et al., 1988), viral infections (Murray, 1976; Wang et al., 2020), poor lifestyle habits (Ascherio et al., 2012), obesity (Jesús et al., 2016), dietary habits, and so on. Growing empirical evidence suggests that nutrition might impact the systemic immune response in the context of disease and autoimmunity (Alter et al., 1974; Hamza et al., 2015).

Therefore, we wanted to test the effect of *Acer truncatum* oil on demyelinating diseases by means of dietary addition. *Acer truncatum* is a native woody plant widely distributed in China. The main components of vegetable oils are fats consisting of straight-chain fatty acids and glycerides. Monoacylglycerol lipase (ML) and Adipose triglyceride lipase (ATGL) in the body promote the breakdown of these fats into fatty acids and glycerol (Tardelli, 2020; Li et al., 2021). The fatty acid composition of *Acer truncatum oil* we use mainly consists of 12 types of fatty acids: palmitic, stearic, oleic, linoleic, linolenic, arachidic, cis-8-eicosenoic, eicosadienoic, erucic, erucic, lignoceric, NA (Qiao et al., 2018).

Clinical studies showed that the type of fatty acid intake affects the course of the disease (Hu et al., 2002). As an indispensable part of people’s daily diet, fatty acids have increasingly become the focus of researches (Manson et al., 2020; Farukhi et al., 2021; Hyon and Han, 2021). n-3 fatty acids are one type of long-chain polyunsaturated fatty acids (LCPUFAs). Nuts and deep-sea fish are significant sources of n-3 LCPUFAs for humans. Moreover, α-linolenic acid is the essential n-3 fatty acids, a major dietary n-3 LCPUFA. The body can convert α-linolenic acid to docosahexaenoic acid (DHA) and eicosapentaenoic acid (EPA), which have antagonistic effects on inflammation-promoting arachidonic acid (AA) (Luxwolda et al., 2011). In a diet-related experiment, organismal inflammation was improved by supplementation with n-3 LC-PUFAS (Tian et al., 2019). Oleic acid and cis-11-eicosenoic acid are long-chain monounsaturated fatty acids (LCMUFAs). A prospective study about MS found that the n-9 LCMUFAs were associated with a better prognosis, whereas n-7 LCMUFAs were associated with a worse prognosis (Hon et al., 2011). NA belongs to n-9 fatty acids and is essential for the growth and maintenance of the brain and peripheral nerve tissues. It binds to sphingosine to form sphingomyelin, which is an integral component of myelin (Martínez and Mougan, 2010). Some clinical studies and animal disease models suggest that, on the one hand, NA intake can promote brain development (Fan et al., 2018; Li et al., 2019). The NA level in sphingomyelin of red blood cells from premature infants may reflect NA levels in sphingomyelin of the brain and could thus reflect brain maturity (Babin et al., 1993); On the other hand, NA can relieve the symptoms of some neurodegenerative diseases (Zheng et al., 2017). In addition, an impairment in the provision of NA in demyelinating diseases is indicated, suggesting that dietary supplementation with oils rich in LCMUFAs may be beneficial in such conditions (Sargent et al., 1994).

The oil in *Acer truncatum* oil contains high concentration LCMUFAs, especially NA. So we investigated the effect of *Acer truncatum* oil on myelin content, ultrastructure, and inflammatory markers in the mouse MS model induced by Cuprizone. Our results showed that in mice supplemented with *Acer truncatum* oil in the diet, the remyelination in the corpus callosum was greatly enhanced with increased mature oligodendrocytes (CC1) and myelin basic protein (MBP). Increasingly, the supplementation with *Acer truncatum* oil in the diet reduced the schizophrenia-like behavior in the open field test and the elevated plus-maze compared to the cuprizone recovery group. The results revealed that the diet supplementation with *Acer truncatum* oil improved behavioral abnormalities, oligodendrocyte maturation, and remyelination in the cuprizone model during recovery. This indicates that *Acer truncatum* oil has great therapeutic potential in multiple sclerosis.

## Materials and Methods

### Animals

Eight-weeks-old female C57BL/6 J mice were purchased from the Laboratory Animals Center of Xi’an Jiaotong University (Xi’an, China). Mice were acclimatized for 1 week before the formal experiments. The mice were housed three together in 40 × 25 × 15 cm cages at room temperature (23–25°C) for 12 h of light/dark cycle. Throughout the acclimatization and experimental periods, food and tap water were available *ad libitum*. All experimental procedures were approved by Northwest A&F University and performed according to the guidelines of the National Institutes of health for laboratory animals.

### Diets and Experimental Design

**FIGURE 1:**
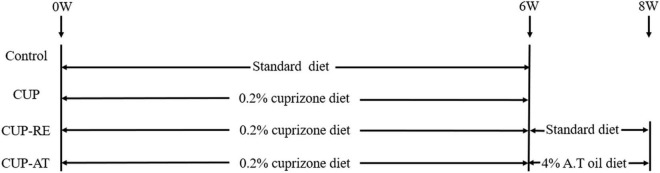
Experimental time course image.

Sixty mice were randomly divided into four groups (*n* = 15 in each group) (As shown in Figure 1): (i) The standard control group (Control): Fed with a basal diet for 6 weeks; (ii) The cuprizone group (CUP) to induce demyelination, cuprizone [bis (cyclohexylidenehydrazide)] (Macklin) at a concentration of 0.2% was mixed into mouse basal diet and fed *ad libitum* for 6 weeks; (iii) The cuprizone recovery group (CUP-RE), in which mice were first fed a basal diet containing 0.2% cuprizone for 6 weeks, then returned to basal diet for 2 weeks; (iv) The *Acer truncatum* oil group (CUP-AT), in which mice were first fed a basal diet containing 0.2% cuprizone for 6 weeks, then returned to the basal diet with 4% *Acer truncatum* oil for 2 weeks. The basal diet contained all the mice’s basic nutrition requirements and was composed of 61% energy substances, 30% protein, 2% fats, and 4% trace elements. *Acer truncatum* oil was added at 4% of the diet.

### Liquid Chromatography-Tandem Mass Spectrometry

#### Sample Preparation and Extraction

10 mg sample was added to the 2 mL centrifugal tube. 1 mL lipid complex solution (Acetonitrile: IPA = 1:1) was added and vortexed for 1 min. Take 10 μL of the previous diluent, add 20 μL of 10 μM internal standard mixed working solution, 970 μL of lipid reconstituted solution, shake for 1 min, and centrifuge at 12,000 g at 4°C for 3 min. 120 μL Supernatant solution was collected for liquid chromatography-tandem mass spectrometry (LC-MS/MS) analysis.

#### HPLC Conditions

The sample extracts were analyzed using an LC-ESI-MS/MS system (UPLC, ExionLC AD; MS, QTRAP^®^ 6500 + System). The analytical conditions were as follows, UPLC: column, Thermo Accucore™C30 (2.6 μm, 2.1 mm × 100 mm i.d.); solvent system, A: acetonitrile/water (60/40 V/V, 0.1% formic acid, 10 mmol/L ammonium formate), B: acetonitrile/isopropanol (10/90 VV/V, 0.1% formic acid, 10 mmol/L ammonium formate); gradient program, A/B (80:20, V/V) at 0 min, 70:30 V/V at 2 min, 40:60 V/V at 4 min, 15:85 V/V at 9 min, 10:90 V/V at 14 min, 5:95 V/V at 15.5 min, 5:95 V/V at 17.3 min, 80:20 V/V at 17.3 min, 80:20 V/V at 20 min; flow rate, 0.35 ml/min; temperature, 45°C; injection volume: 2 μL. The effluent was alternatively connected to an ESI-triple quadrupole-linear iontrap (QTRAP)-MS.

#### ESI-Multiple Sclerosis /Multiple Sclerosis Conditions

LIT and triple quadrupole (QQQ) scans were acquired on a triple quadrupole-linear ion trap mass spectrometer (QTRAP), QTRAP^®^ 6500 + LC-MS/MS System, equipped with an ESI Turbo Ion-Spray interface, operating in positive and negative ion mode and controlled by Analyst 1.6.3 software (Sciex). The ESI source operation parameters were as follows: ion source, turbo spray; source temperature 500°C; ion spray voltage (IS) 5,500 V (Positive), –4,500 V (Neagtive); Ion source gas 1 (GS1), gas 2 (GS2), curtain gas (CUR) were set at 45, 55, and 35 psi, respectively. Instrument tuning and mass calibration were performed with 10 and 100 μmol/L polypropylene glycol solutions in QQQ and LIT modes, respectively. QQQ scans were acquired as MRM experiments with collision gas (nitrogen) set to 5 psi. DP and CE for individual MRM transitions was done with further DP and CE optimization. A specific set of MRM transitions were monitored for each period according to the metabolites eluted within this period.

### Open Field Test

The experimental setup is a square box with an open top (50 cm × 50 cm × 30 cm). The equipment was confirmed to be clean and taste-free and wiped with 75% ethanol every time after testing a mouse. A 60 W light bulb was positioned 100 cm above the arena’s base and provided the only illumination source in the testing room. The test time for each mouse was 5 min. During the experiment, the total distance and the time, distance, and frequencies in the central area were recorded.

### Elevated Plus Maze

The elevated plus maze (EPM) device is 70 cm high above the ground, consists of two open arms (dimensions: 35 × 6 cm), two closed arms (dimensions: 35 × 6 × 14 cm) and a central region (dimensions:6 × 6 cm). The equipment was confirmed to be clean and taste-free and wiped with 75% ethanol every time after testing a mouse. A 60 w light hung at 100 cm above the center of the unit. Put the animal in the center of the equipment with its head facing the open arm. The duration of the test was 5 min. During the test. During the test, the time, distance, and frequencies in open arms were recorded.

### Forced Swim Test

Animals were forced to swim in a glass chamber (dimensions: 30 cm height × 15 cm diameters) containing 25°C water 20 cm deep, avoiding mice touching the bottom with their feet. The animals were allowed to swim till exhaustion. The test remained for 5 min, recording the immobility time in the last 4 min of the 5 min. After 5 min, the mice were removed from the glass chamber, dried with a towel, and placed back in their home cage to dry. The water in the swim tank was changed between animals.

### Tail Suspension Test

The mice were suspended on the support by adhesive tape placed approximately 1 cm from the tip of the tail. The distance from the mouse’s nose to the tabletop was 10 cm (Tovchiga et al., 2018). After initial vigorous activities, the animal assumes an immobile posture, and the period of immobility during the last four min of the five min observation period was recorded. All ratings were made by an observer unaware of the animal treatments.

### Rota-Rod Test

A Rota-Rod treadmill was used to test the motor coordination and endurance of animals (Franzone and Reboani, 1977). A longer time remaining on the rolling treadmill was equated with better coordination and endurance. Mice were trained at 18 rpm/min for 3 min on two consecutive days at 9 a.m. On the third day, mice were tested at 40 rpm/min. Then the timer automatically stopped when the animal fell off, with a maximal cutoff time of 300 s.

### Myelin Staining by Luxol Fast Blue

Mice were anesthetized by sodium pentobarbital transcardially and perfused with 0.9% physiological saline followed by 4% paraformaldehyde (PFA) in 0.1 M phosphate buffer (PB, pH = 7.4) for 15 min. After perfusion, the brains were removed from the skull, then postfixed in 4% PFA overnight. The fixed brains were cut into 3 mm-thick blocks and placed in a container with 20% sucrose in 0.1 M PB until the tissue sank to the container’s bottom. The tissue was then transferred to a 30% sucrose in 0.1 MPB for 48 h. The brains were embedded in the OCT compound (Tissue-Tek^®^, Sakura) and coronally sliced at a thickness of 20 μm using a Freezing Microtome (POLAR DM, Sakura, Japan).

The sections were stained with luxol fast blue (LFB) (Sigma, United States) solution (0.01% LFB in 95% ethanol with 10% acetic acid) overnight at 56°C. After being rinsed in 95% ethanol for 3 min, the sections were postfixed in 1% lithium carbonate (Macklin, CHINA) solution for 10 s and subsequently rinsed in 70% ethanol for 10 s. This step was repeated until a clear differentiation between gray and white matter staining. Eventually, sections were rinsed in aqua bidest and mounted with glycerol jelly mounting medium (Solarbio, CHINA). Images were taken using a universal research motorized microscopy (Ni-U, Sakura, JAPAN).

### Immunofluorescence

Mice were anesthetized and perfused with 4% PFA as above. The fixed brains were cut into 50 μm coronal sections using a vibration microtome (VT1000S, Leica, Germany). The sections were incubated with the primary antibody in a blocking solution containing 2% bovine serum albumin (BSA, Germany), 0.3% Triton X-100 (Solarbio, China), 1% normal goat serum (Vector Laboratories, United States) in PB at 4°C overnight. Rinsing 10 min three times with PB, the slices were incubated in secondary antibody at room temperature in the dark for 4 h. After three rinses with PB, sections were counterstained with DAPI and then covered with the fluorescent mounting medium (Dako, United States), and images were captured with a fluorescence microscope (Axio Obser Z1, Zeiss, Germany). The following antibodies were used: mouse anti-adenomatous polyposis coli (CC1, 1:500, Abcam, AB16794, England), Rat anti-MBP (1:500, Abcam, AB7349, England), rabbit anti-GFAP (1:1,000, Abcam, AB7260, England), rabbit anti-Iba1 (1:1,000, Abcam, AB178846, England).

### Transmission Electron Microscopy

Mice were anesthetized by sodium pentobarbital and transcardially and perfused with 0.9% physiological saline, followed by 4% formaldehyde containing 0.1% glutaraldehyde (BASF, Germany) in 0.1 M PB for 15 min. After perfusion, the brains were removed from the skull, then postfixed in paraformaldehyde for 24 h in 4% PFA containing 0.1% glutaraldehyde and coronally cut into sections with a thickness of 200 μm by using a vibration microtome (VT1000S, Leica, Germany). The slices were postfixed with 1% osmium acid for 30 min, then rinsed several times with 0.1 M PB. The sections were dehydrated with increasing concentrations of alcohol, then placed in a 1:1 solution of ethanol and acetone, and 100% acetone, and permeated, respectively, with a mixture of acetone and Epon 812 resin (MNA, EROK, DDSA, PMP-30), and Epon 812 overnight at room temperature. The slices were embedded with Epon 812 between the slide and the cover glass coated with the mold parting compound, then placed into an oven. The program of the oven was set as follows: 37°C, 2 h; 45^°^C, 2 h; 60°C, 48 h. After polymerization, the sections were removed from slides and cover slides and washed with 75% alcohol to clean the mold parting compound. The corpus callosum of the brains was cut into 1 × 1 mm square tissue blocks under the stereomicroscope (M165 FC, Leica, Germany). The tissue blocks were embedded into the capsule with Epon 812 and placed again into an oven for polymerization. After polymerization, the capsules were trimmed under the trimming machine (EM TRIM2, Leica, Germany). The tissues were cut into 70 nm slices with diatom trimming blades and the ultramicrotome (EM UC7, Leica, Germany). After being stained with uranyl acetate and lead citrate, slices were observed and photographed under the transmission electron microscope (Tecnai G2 Spirit Bio, United States).

## Results

### Main Ingredients of *Acer truncatum* Oil

**FIGURE 2:**
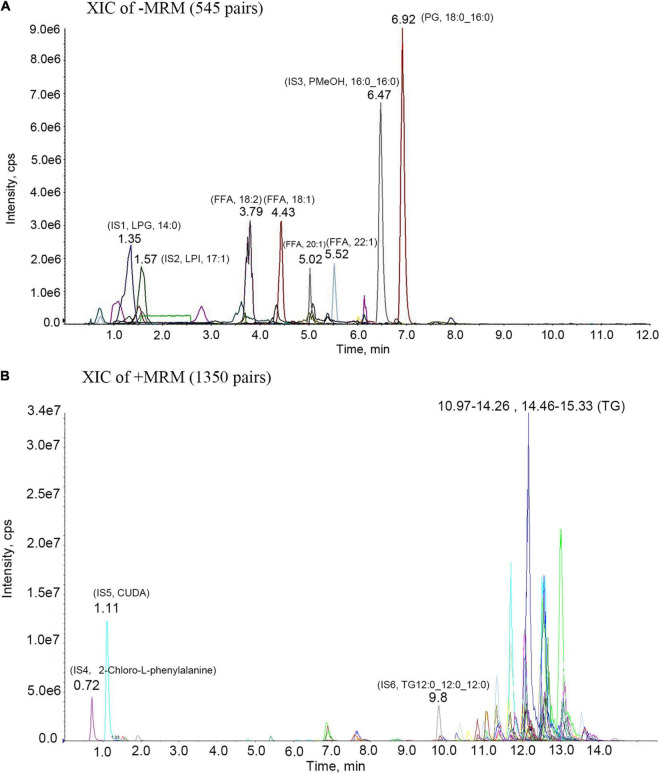
MRM metabolite assay multi-peak chart. IS1-IS6 are all internal standard; **(A)**-MRM metabolite assay multi-peak chart. 3.79- (FFA, 18:2)- Linoleic acid; 4.43- (FFA, 18:1)- Oleic acid; 5.02- (FFA, 20:1)- Eicosenoic Acid; 5.52- (FFA, 22:1)- Erucic acid; 6.92- (PG, 18:0_16:0)- Phosphatidyl Glycerols (18:0_16:0); **(B)** + MRM metabolite assay multi-peak chart. 11.33- (TG,16:0_18:2_18:2)- Triglyceride (16:0_18:2_18:2); 11.7- (TG,18:2_18:2_20:1)- Triglyceride (18:2_18:2_20:1); 12.15- (TG, 18:2_18:2_22:1)- Triglyceride (18:2_18:2_22:1); 13- (TG,18:1_18:1_22:1)- Triglyceride (18:1_18:1_22:1).

We performed LC-MS/MS on methionite maple oil and analyzed the results by MRM. As shown in Figure 2 and Table 1, we collated and analyzed these lipid contents: Triglyceride (TG), 85.91%; Free Fatty Acid (FFA), 6.69%; Diacylglycerol (DG), 6.11%; Monoacylglyceride (MAC), 0.37%; Phosphatidylserine (PS), 0.30%; Phosphatidylcholine (PC), 0.24%; Lysophosphatidic Acids (LPA), 0.12%; Phosphatidic Acid (PA), 0.10%.

**TABLE 1:** Main ingredients of *Acer truncatum* oil.

Ingredients	TG	FFA	DG	MAC	PS	PC	LPA	PA
**Proportion**	85.91%	6.69%	6.11%	0.37%	0.30%	0.24%	0.12%	0.10%

### *Acer truncatum* Oil Recovered Demyelination Induced by Cuprizone

**FIGURE 3:**
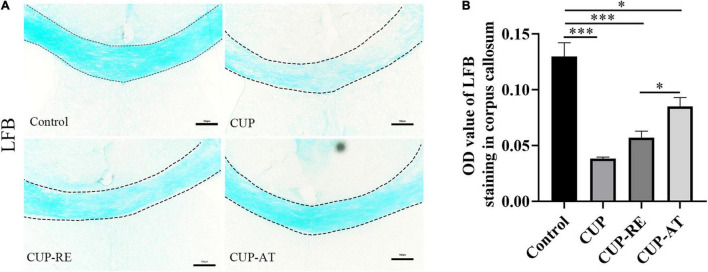
Administration of CUP induced demyelination of the corpus callosum and dietary *Acer truncatum* oil promoted remyelination in the corpus callosum. **(A)** LFB staining in the corpus callosum. The corpus callosum in control group was densely and regularly labeled with LFB, whereas the LFB staining in mice administrated with CUP became light and irregular. After dietary *Acer truncatum* oil, the LFB staining recovered almost like that in control group. **(B)** Quantitative analysis of *OD* value of LFB in the corpus callosum. Acute CUP administration significantly reduces the *OD* value of LFB. Dietary *Acer truncatum* oil rescued the reduction of *OD* value of LFB. *n* = 3–4/group. Scale bar: 100 μm. Data were expressed as means ± SEM. Differences between groups were expressed as *p < 0.05, ^***^p < 0.001.

The hallmark of MS is the demyelination of the nervous system, especially in the white matter of the brain. To evaluate demyelination and remyelination in mice, the coronal sections of the corpus callosum were stained with LFB, which specifically binds to sphingomyelin and visualizes the myelin sheath. In control group, the corpus callosum was densely and regularly labeled by LFB, whereas the LFB staining in mice administrated with CUP became light and irregular (Figure 3A). The *OD* value of LFB in the corpus callosum of mice administrated with CUP was significantly decreased compared with control group (Figure 3B, *p* < 0.001). Interestingly, supplementation in diet with *Acer truncatum* oil increased the *OD* value of LFB in the corpus callosum (Figures 3A,B, *p* < 0.05). These results indicate that *Acer truncatum* oil promotes remyelination in the CUP model.

### *Acer truncatum* Oil Promote Remyelination in the Corpus Callosum by Accelerating the Proliferation of Mature Oligodendrocytes

**FIGURE 4:**
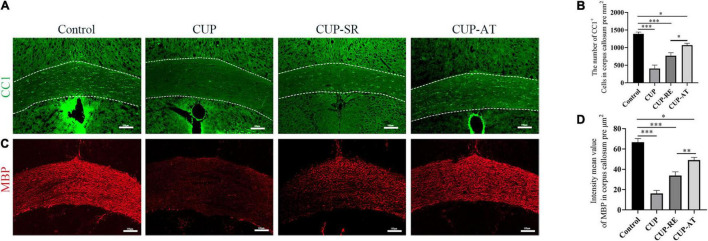
CUP affects the maturation of oligodendrocytes in the corpus callosum, supplementation with *Acer truncatum* oil accelerated the maturation of oligodendrocytes and promoted remyelination. **(A)** CC1 immunofluorescence staining was performed in coronal slices of the corpus callosum in different groups. In control group, the CC1-positive mature oligodendrocytes in the corpus callosum were densely arranged, whereas the number of mature oligodendrocytes in the corpus callosum administrated with CUP significantly reduced. **(B)** Quantitative analysis of CC1-positive cells in the corpus callosum. The density of CC1-positive cells was significantly reduced in the CUP group. Dietary *Acer truncatum* oil significantly increased the number of CC1-positive cells. *n* = 4–6/group. **(C)** Fine MBP-positive fibers were well observed in the corpus callosum from control mice. In contrast, the mice exposed to CUP for 6 weeks showed widespread myelin breakdown. After dietary *Acer truncatum* oil, the MBP immunofluorescence staining recovered almost like that in control group. **(D)** Quantitative analysis of fluorescence intensity means the value of MBP in the corpus callosum. MBP expression in the corpus callosum was significantly decreased after CUP exposure, whereas *Acer truncatum* oil supplementation significantly increased MBP expression. *n* = 8/group. Scale bar: 100 μm. Data were expressed as means ± SEM. Differences between groups were expressed as *p < 0.05, ^**^p < 0.01, ^***^p < 0.001.

Myelin sheath is composed of consecutive layers of the plasma membrane that enwrap axons in the nervous system (Wilkins et al., 2003). Mature oligodendrocytes are responsible for the formation and maintenance of the myelin sheaths in the central nervous system. To clarify the changes in mature oligodendrocytes, immunohistochemistry was used to evaluate CC1 (the specific marker of mature oligodendrocytes) in the corpus callosum. The results showed that compared with control group, the number of CC1-labeled oligodendrocytes in the corpus callosum was significantly lower in the mice administrated with CUP (Figures 4A,B, *p* < 0.001). There was a significant increase in the number of mature oligodendrocytes in the corpus callosum of mice given *Acer truncatum oil* compared to CUP-RE group (Figures 4A,B, *p* < 0.05). Then, immunohistochemistry was used to evaluate MBP (the marker of myelin sheath) in the corpus callosum, which is used to detect the protein component of myelin and is sensitive to the loss of myelin, making it suitable for monitoring remyelination (Katsara et al., 2008; Liu et al., 2019). The results are consistent with the above (Figure 3), fine MBP-positive fibers were well visualized in the corpus callosum from control mice. In contrast, the mice exposed to CUP for 6 weeks showed widespread myelin breakdown (Figures 4C,D, *p* < 0.001). Moreover, when CUP was withdrawn from the diet for 2 weeks, compared to the CUP-RE group, the MBP labeling was observed significantly in CUP-TA groups (Figures 4C,D, *p* < 0.01). This indicates that *Acer truncatum* oil promotes remyelination by accelerating the proliferation of mature oligodendrocytes.

### *Acer truncatum* Oil Effectively Attenuated Astrocytic Activation Induced by Cuprizone

**FIGURE 5:**
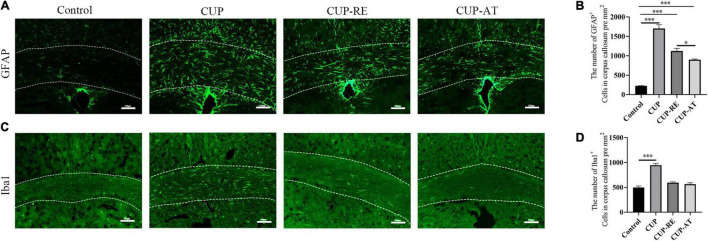
Supplementation with *Acer truncatum* oil inhibited the activation of microglia and astrocytes after administration with CUP. **(A)** GFAP immunofluorescent staining (green) was performed in coronal slices of the corpus callosum. In control mice, astrocytes were only sporadically observed. CUP induced an increase in the number of astrocytes and cytosolic hypertrophy, which were attenuated by *Acer truncatum* oil supplementation. **(B)** Quantitative analysis of GFAP-positive cells in the corpus callosum. CUP administration significantly increases the number of astrocytes, dietary supplementation of *Acer truncatum* oil significantly decreased the number of GFAP-positive cells. *n* = 4/group. **(C)** Iba1 immunofluorescent staining (green) was performed in coronal slices of the corpus callosum. In control mice, microglia were only sporadically observed. CUP induced an increase in the number of microglia, which were attenuated by *Acer truncatum* oil supplementation. **(D)** Quantitative analysis of Iba1-positive cells in the corpus callosum. CUP administration significantly increased the number of astrocytes, supplementation of *Acer truncatum* oil decreased the number of astrocytes cells. However, the difference was not significant. *n* = 4–6/group. Data were expressed as means ± SEM. Differences between groups were expressed as *p < 0.05, ^***^p < 0.001.

Astrocytes are essential for the regeneration of oligodendrocytes and myelin after CUP-induced demyelination (Yamazaki et al., 1993). To detect the expression of astrocytes, glial fibrillary acidic protein (GFAP) immunofluorescence staining was performed on each group of corpus callosum (Figure 5A). Significant increase in the number of astrocytes in CUP group as compared with control group (Figure 5B, *p* < 0.001). However, following 2 weeks of dietary *Acer truncatum* oil, GFAP positive cells significantly decreased as compared with that in CUP group (Figure 5B, *p* < 0.05). Astrocytes regulate myelin clearance through recruitment of microglia during cuprizone-induced demyelination (Thomas et al., 2013). To evaluate the microglia population in the corpus callosum, Iba1 immunostaining was performed in the different groups (Figure 5C). Iba1 positive cells significantly increased in CUP group as compared to that in control group (Figure 5D, *p* < 0.001). No significant difference in the CUP-AT group compared to the CUP-RE group. The above results indicate that CUP administration induced an increase of astrocyte and *Acer truncatum* oil supplementation inhibits astrocyte changes in the corpus callosum.

### Supplementation of *Acer truncatum* Oil Can Be of Advantage to Recover the Damage of Axonal and Myelin in the Cuprizone Mouse Model

**FIGURE 6:**
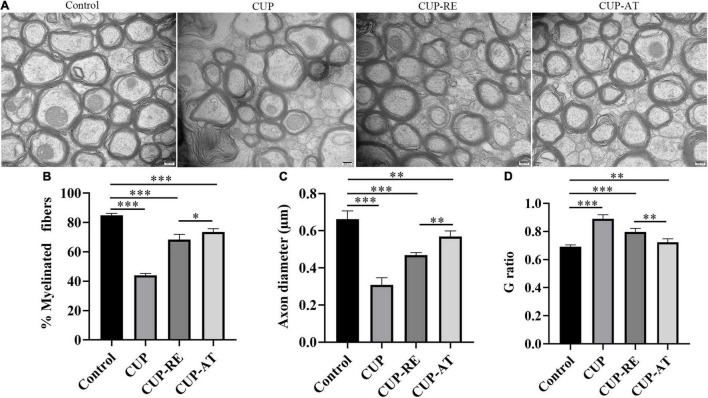
*Acer truncatum* oil supplementation can be of advantage to recover the damage of axonal and myelin in the MS mouse model. **(A)** The TEM photomicrographs were taken from coronal sections of the corpus callosum, healthy myelin sheaths with normal and intact structures were observed of the corpus callosum in control group, whereas disintegration of neural fibers was found in CUP mice. *Acer truncatum* oil supplementation was efficient in the recovery of myelin sheaths from pathological morphology, in comparison to CUP-RE group mice, in which lower electron density and less well-defined myelin sheaths was found. **(B)** Quantitative analysis of the myelinated fibers. The percentage of the myelinated fibers in the corpus callosum of mice administrated with CUP significantly decreased compared with that in control group. Dietary *Acer truncatum* oil supplementation significantly increased in axon diameter as compared to that in the CUP-RE group. **(C)** Quantitative analysis of the axon diameter. Axon diameter in mice administrated with CUP significantly decreased compared with that in control group, Axon diameter after dietary *Acer truncatum* oil supplementation significantly increased as compared to that in the CUP-RE group. **(D)** Quantitative analysis of the G ratio. The G ratio in CUP mice was significantly increased compared with that in control group, after dietary *Acer truncatum* oil treatment, the G ratio was significantly reduced compared with the CUP-RE group. *n* = 9/group. Scale bar: 200 nm. Data were expressed as means ± SEM. Differences between groups were expressed as *p < 0.05, ^**^p < 0.01, ^***^p < 0.001.

To validate the effect of *Acer truncatum* oil on myelin pathology, the ultrastructure of the corpus callosum was also investigated with transmission electron microscopy (TEM). Healthy myelin sheaths with normal and intact structures were observed under TEM in control group. In contrast, the disintegration of neural fibers was found in mice administrated with CUP. Supplementation of *Acer truncatum* oil was efficient in remyelination, in comparison to CUP-RE group mice, in which lower electron density and less well-defined myelin sheaths was observed (Figure 6A). Quantitative data showed that the percent of the myelinated fibers was significantly decreased in CUP group in comparison to control (Figure 6B, *p* < 0.001). Importantly, supplementation of *Acer truncatum* oil caused a significant increase in the percentage of myelinated fibers as compared to that in CUP-RE group (Figure 6B, *p* < 0.05). In addition, axon diameter was significantly decreased in CUP group in comparison to control (Figure 6C, *p* < 0.001), while supplementation of *Acer truncatum* oil caused a significant increase in axon diameter as compared to CUP-RE group (Figure 6C, *p* < 0.01). Axon diameter and myelin thickness were also used to calculate the G ratio (axon diameter divided by myelinated fiber diameter) as an indicator of remyelination. Quantification analysis by G ratio showed that the myelin thickness significantly decreased in CUP group compared to control group (Figure 6D, *p* < 0.001) and increased after *Acer truncatum* oil supplementation compared to CUP-RE group (Figure 6D, *p* < 0.01). These results indicate that *Acer truncatum* oil supplementation can be of advantage to recover the damage of axonal and myelin in the MS mouse model.

### Dietary Supplementation of *Acer truncatum* Oil Improved the Recovery of Cuprizone-Induced Behavioral Changes in Mice

**FIGURE 7:**
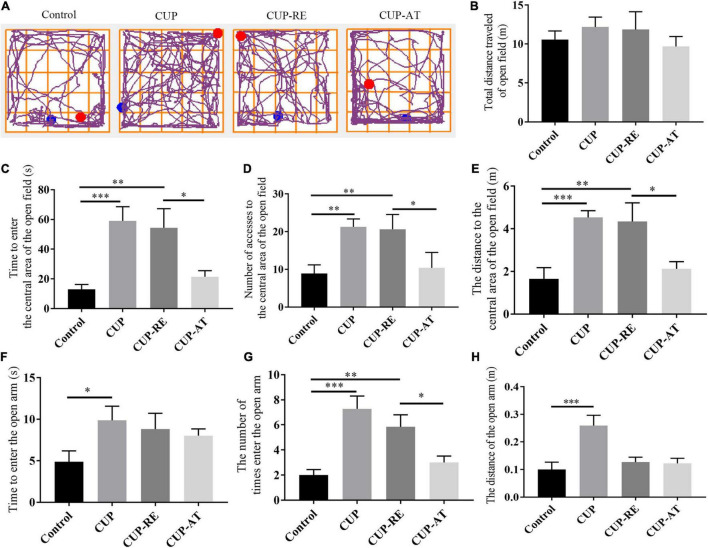
Dietary supplementation of *Acer truncatum* oil promoted the recovery of CUP-induced behavioral changes in mice. **(A)** Representative trajectory images of each group of OFT. **(B–E)** No significant difference in the total distance was found in the OFT between the groups. The activity time **(C)**, number **(D)** and accumulated distance **(E)** of the CUP-exposed mice in the central area of the OFT were significantly higher than that of mice in control group; After CUP-exposure, 2-week *Acer truncatum* oil dietary supplementation promoted the recovery of CUP-induced behavioral changes compared to that in mice of CUP-RE group. *n* = 8–12/group. **(F–H)** CUP-exposed mice showed significantly higher activity time **(F)** number **(G)** and distance traveled **(H)** in the open arm of EMP. Dietary supplementation of *Acer truncatum* oil significantly reduced the number of mice entering the open arm compared with that in CUP-RE. *n* = 6–7/group. Data were expressed as means ± SEM. Differences between groups were expressed as *p < 0.05, ^**^p < 0.01, ^***^p < 0.001.

CUP-induced behavioral changes include ataxia, anxiety and cognitive deficits, which are thought to be associated with demyelination (Sen et al., 2019). Therefore, to investigate the effects of dietary supplementation with *Acer truncatum* oil on cognitive impairment in demyelinated mice, we conducted a series of behavioral experiments in four groups of mice. We examined the neurobehavior of the mice administrated with CUP by using open field test (Figure 7A). No significant difference in the total distance among the groups was found (Figure 7B). However, cuprizone administration significantly increased the time (Figure 7C, *p* < 0.001), number (Figure 7D, *p* < 0.01), and distance (Figure 7E, *p* < 0.001) in the center area (Figures 7C–E, *p* < 0.001). Supplementation of *Acer truncatum* oil to the diet can improve abnormal emotional behavior caused by myelin damage: Mice in CUP-AT group had a significantly lower activity time (Figure 7C, *p* < 0.05), number (Figure 7D, *p* < 0.05), and accumulated distance (Figure 7E, *p* < 0.05) in the central area of the open field compared to that of mice in the CUP-RE group. In EPM, CUP administration significantly increased the time (Figure 7F, *p* < 0.05), number (Figure 7G, *p* < 0.001), and distance (Figure 7H, *p* < 0.001) spent in the open arm. Dietary supplementation of *Acer truncatum* oil significantly reduced the number of entering the open arm (Figure 7G, *p* < 0.05). These data suggest that dietary supplementation of *Acer truncatum* oil promoted the recovery of CUP-induced abnormal behavior in mice.

**FIGURE 8:**
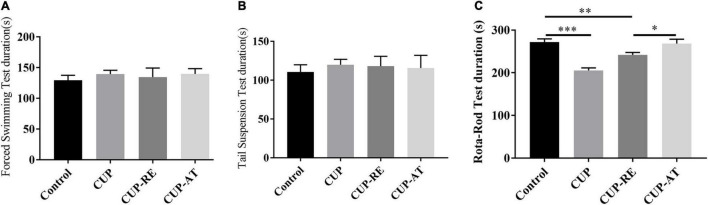
Dietary supplementation of *Acer truncatum* oil improved the recovery of CUP-induced physical coordination in mice. **(A)** The immobility time of the last 4 min of the 5 min of FST. *n* = 8–15/group. **(B)** The immobility time of the last 4 min of the 5 min of TST. *n* = 6–10/group. **(C)** Dietary supplementation of *Acer truncatum* oil significantly improved the recovery of CUP-induced Rota-rod changes in mice. *n* = 8–12/group. Data were expressed as means ± SEM. Differences between groups were expressed as *p < 0.05, ^**^p < 0.01, ^***^p < 0.001.

To investigate the effects of dietary supplementation of *Acer truncatum* oil on endurance and limb coordination in demyelinated mice, we performed several behavioral tests, includes forced swim test (FST) and tail suspension test (TST) for endurance, Rota-rod test for endurance and coordination. No significant difference among the groups in the results of the FST (Figure 8A) and the TST (Figure 8B) was found. However, the dietary supplementation of *Acer truncatum* oil improved the coordination ability of mice in the Rota-rod test for endurance and coordination (Figure 8C, *p* < 0.05). These data suggest that dietary supplementation of *Acer truncatum* oil promoted the recovery of CUP-induced physical coordination in mice.

## Discussion

MS involves white matter abnormalities and neuroinflammation in the brain. Because of these two pathological features and the concurrent behavioral changes, the CUP-exposed mouse has provided an animal model of demyelination and remyelinating, as well as to examine the neuroinflammation and oligodendrocyte dysfunction hypotheses of MS. In the present study, CUP exposure for 6 weeks induced dramatic decrease in oligodendrocyte and salient demyelination in the corpus callosum, accompanied by behavioral deficits indicative of motor, anxiety, and cognitive behaviors. All these deficits replicated the findings in previous studies by the others (Franco-Pons et al., 2007; Matsushima and Morell, 2010). More significantly, dietary supplementation of *Acer truncatum* oil for 2 weeks promoted the behavioral recovery of CUP-exposed mice and accelerated the remyelinating process in mice after CUP withdrawal. In addition, dietary supplementation of *Acer truncatum* oil inhibited microglia and astrocyte activation.

*Acer truncatum* is a woody tree species that produce seeds containing high concentration oil with valuable fatty acids. Previous studies have suggested that *Acer truncatum* oil would be an excellent edible oil (Wang et al., 2006; Peng et al., 2017). Our test results found that methionite maple oil consisted mainly of TG, FFA and DG. Dietary TG are emulsified by bile acids within the intestinal lumen following their hydrolysis primarily by pancreatic lipase (Carey and Hernell, 1992; Armand et al., 1996; Lowe, 2002), which yields sn-2-monoacylglycerols and free FFA as products; In addition to this, ATGL catalyzes the hydrolysis of TG, releasing DG. This lipid is further hydrolyzed by hormone sensitive lipase (HSL), releasing monoacylglycerol (MG). Monoacylglycerol lipase (MGL) mediates the breakdown of MG into FFA and glycerol (Alves-Bezerra and Cohen, 2017; Grabner et al., 2021).

Whether consumed directly through Acer truncatumoil or through ingestion of TG for its own breakdown, the body ends up with a large amount of LCPUFAs, LCPUFAs are not only a healthier fatty acid, but also have been shown to be effective in the treatment of many diseases, as explained in the section “Introduction.” TG is broken down by the body into FFA and also into DG, which reduces postprandial hyperlipidemia and glycated hemoglobin and increases energy expenditure (Tada, 2004). DG is a key lipid intermediate linking nutrient excess to the antagonism of insulin signaling (Shmueli et al., 1993), and this molecule has been shown to accumulate in muscles obtained from insulin-resistant rodents and humans (Montell et al., 2001; Itani et al., 2002). In neural and non-neural cells insulin-mediated stimulation of phospholipase C (PLC) results in degradation of membrane phosphatidylinositol 4,5-bisphosphate (PtdIns-4,5-P_2_) leading to the formation of DAG and inositol 1,4,5-trisphosphate (Ins-1,4,5-P_3_). DG stimulates various isoforms of protein kinase C (PKC) isoforms, and Ins-1,4,5-P_3_ mobilizes calcium from intracellular stores. No information is available on the effect of DG ingestion on signal transduction processes in the brain. Similarly, levels of DG in brains of metabolic syndrome patients have not been determined. However, ischemic injury, AD and depression are accompanied by the breakdown of neural membrane phospholipids and alterations in levels of DG (Farooqui, 2011). mTOR and cPKC, which are activated by PA and DG, respectively, are involved in the DGKβ-induced neurite induction and branching. It has also been shown that the products of DG breakdown *via* diacylglycerol kinase are an important component of the phospholipid bilayer, which may also be an important source of myelin production (Tu-Sekine and Raben, 2011; Kim et al., 2014). We therefore conjecture that *Acer truncatum* oil could be effective in demyelinating diseases.

The corpus callosum connects the left and right cerebral hemispheres and is the largest white matter structure in the brain. It is primarily responsible for transmitting sensory, motor and cognitive information between the regions (Grieve et al., 2007; Bénézit et al., 2015). Several studies on human diseases have found brain white matter fiber abnormalities and corpus callosum alterations in patients with axonal and demyelinating diseases (Rotarska-Jagiela et al., 2008; Saia-Cereda et al., 2015). This implies that cerebral white matter lesions and corpus callosum abnormalities may be closely related to the pathogenesis of MS. Previous studies have reported decreased expression of myelin-related protein levels in patients with demyelination and cerebral white matter, such as MBP (Burbaeva et al., 2006; Parlapani et al., 2009). The present study indicated that CUP exposure led to significant demyelination and decrease in MBP expression in the corpus callosum of mice, while supplementation in diet with *Acer truncatum* oil increased remyelination and MBP expression in the corpus callosum of CUP administrated mice. Moreover, similar results in histological staining and TEM were found, showing that *Acer truncatum* oil promote remyelination in the CUP model.

Mature oligodendrocyte is essential for remyelination. In the present study, CUP administration induced demyelination accompanied by decrease in CC1-labeled mature oligodendrocytes in the corpus callosum. *Acer truncatum* oil supplementation elevated the number of CC1-labeled mature oligodendrocytes. This suggests that *Acer truncatum* oil facilitates remyelination. This may be one of the mechanisms by which *Acer truncatum* oil alleviates demyelination and white matter lesions induced by CUP. Mature oligodendrocytes as opposed to many cell types in the central nervous system have pools of readily available progenitor cells called Oligodendrocyte precursor cells (OPCs). OPCs undergo a series of defined steps to differentiate into mature oligodendrocytes (Li et al., 2005). Mature oligodendrocyte is a major type of neuroglial cell in the central nervous system that have the extraordinary ability to wrap their cytoplasm membrane around the axons of neurons to form myelin (Kuhn et al., 2019). However, some diseases such as MS, stroke, and spinal cord injury cause degeneration of mature oligodendrocytes and induced damage of myelin sheath (Emery, 2010; Zuchero and Barres, 2013). In CUP administrated animals that undergo demyelination, increase in OPCs and dramatic decrease in mature oligodendrocytes were observed in the corpus callosum, suggesting a blockade of OPCs differentiation into mature oligodendrocytes (Zhang et al., 2014; Abulimiti et al., 2015), *Acer truncatum* oil promote remyelination by accelerated the differentiation of OPCs to mature oligodendrocytes.

The immune system plays pivotal role in remyelination. Madadi’s research found that microgliosis and astrogliosis are two important pathological events in the CUP-induced demyelination model, and involved in MS pathologenesis by increasing oligodendrocyte apoptosis, oxidative stress, neuroinflammation, and decreasing OPCs differentiation (Madadi et al., 2019). Astrocytes become reactive in response to neuroinflammatory stimuli, and GFAP expression is upregulated during the process of astrogliosis (Nair et al., 2008). Several studies showed that activated astrocytes promote demyelination, prevent remyelination, exert toxic effects on oligodendrocytes by secreting proinflammatory cytokines (Elena et al., 2005; Serbinek et al., 2010). The present study found that administration with CUP induced prominent astrocyte activation and demyelination deformation in the corpus callosum, which was reversed by *Acer truncatum* oil supplementation in diet. In addition, we found that microglia were remarkably activated after CUP ingestion for 6 weeks. The increase in microglial cells induced by CUP was attenuated by supplementation of *Acer truncatum* oil, indicating that *Acer truncatum* oil plays a protective role in microglia mediated demyelination. The presence of microglia in every step of the evolving white matter lesion suggests a role in clearing the environment of growth inhibitory molecules and expression of growth beneficial molecules (Takahashi et al., 2008; Choi et al., 2016).

It has been confirmed that motor dysfunction is a crucial problem in patients with MS (Coghe et al., 2015). CUP mouse model shows severe motor dysfunction (Li et al., 2015) and schizophrenia-like activity (Liu et al., 2017). By performing a series of behavioral experiments, we have come to the same conclusion. The abnormal behaviors, demyelination, and oligodendrocyte loss observed in CUP administrated mice suggest a conjunction between white matter damage and behavioral disorder symptoms (Li et al., 2015; Okuliarova et al., 2016). In the present study, we found that dietary supplementation with Acer truncatum oil could promote the production of mature oligodendrocytes by inhibiting the proliferation of astrocytes, thereby promoting myelin regeneration and improving behavioral abnormalities caused by CUP in mice. However, direct evidence for these findings is still lacking. Future studies will focus on how *Acer truncatum* oil inhibits astrocytes, how mature oligodendrocytes increase, and through which molecular mechanisms myelin regeneration is promoted.

In conclusion, the present study demonstrates that dietary supplementation of *Acer truncatum* oil improved abnormal behaviors by promoting remyelination in mice.

## Conclusion

In conclusion, the dietary supplementation of *Acer truncatum* oil during the recovery phase of CUP-induced demyelination may have myelin restoration effect and alter schizophrenic-like behavior. Further isolation of active ingredients from *Acer truncatum* oil should be carried out in the future to verify the specific components at play.

## Data Availability Statement

The raw data supporting the conclusions of this article will be made available by the authors, without undue reservation.

## Ethics Statement

The animal study was reviewed and approved by the Northwest A&F University.

## Author Contributions

YX designed the study, analyzed the data, and drafted the manuscript. XZ, WY, ZZ, EC, YW, CL, JP, QY, XC, and SZ selected the studies, extracted the data, and analyzed the data. All authors approved the final manuscript.

## Conflict of Interest

The authors declare that the research was conducted in the absence of any commercial or financial relationships that could be construed as a potential conflict of interest.

## Publisher’s Note

All claims expressed in this article are solely those of the authors and do not necessarily represent those of their affiliated organizations, or those of the publisher, the editors and the reviewers. Any product that may be evaluated in this article, or claim that may be made by its manufacturer, is not guaranteed or endorsed by the publisher.
